# Membrane interaction of retroviral Gag proteins

**DOI:** 10.3389/fmicb.2014.00187

**Published:** 2014-04-29

**Authors:** Robert A. Dick, Volker M. Vogt

**Affiliations:** Department of Molecular Biology and Genetics, Cornell University, IthacaNY, USA

**Keywords:** HIV-1, RSV, lipid, assembly, liquid ordered, plasma membrane

## Abstract

Assembly of an infectious retroviral particle relies on multimerization of the Gag polyprotein at the inner leaflet of the plasma membrane. The three domains of Gag common to all retroviruses – MA, CA, and NC – provide the signals for membrane binding, assembly, and viral RNA packaging, respectively. These signals do not function independently of one another. For example, Gag multimerization enhances membrane binding and is more efficient when NC is interacting with RNA. MA binding to the plasma membrane is governed by several principles, including electrostatics, recognition of specific lipid head groups, hydrophobic interactions, and membrane order. HIV-1 uses many of these principles while Rous sarcoma virus (RSV) appears to use fewer. This review describes the principles that govern Gag interactions with membranes, focusing on RSV and HIV-1 Gag. The review also defines lipid and membrane behavior, and discusses the complexities in determining how lipid and membrane behavior impact Gag membrane binding.

## INTRODUCTION

In enveloped viruses the common function of the viral membrane is to help protect the genome. Having a membrane poses two challenges for the virus. First, the genome, in the form of a nucleocapsid, must acquire its membrane from the appropriate cell compartment that will allow virus release into the environment. Second, upon infection of a target cell, the nucleocapsid must escape the membrane surface. The latter process is mediated by viral surface glycoproteins that act as fusion machines. The former process for some viruses takes place after assembly of the nucleocapsid, while in other viruses occurs concomitantly with assembly. For retroviruses, the topic of this review, assembly typically is coupled with acquisition of the membrane, although with some exceptions.

For most retroviruses, expression of the single internal structural protein, Gag, is sufficient for assembly and budding of immature virus-like particles (VLPs) from the plasma membrane (PM). That Gag can seek out the PM and bud in diverse cell types – for example for HIV-1 in mammalian cells as well as in avian cells and insect cells – implies that Gag recognizes general features of the PM. Several exceptions to this generalization are well known. Spuma (foamy) viruses require expression of the viral envelope glycoprotein for budding to occur, and the specific interaction between Gag and Env that underlies this finding has recently been characterized (Goldstone et al., 2013). In this case the immature but not yet enveloped virus particle is assembled near the microtubule organizing center (MTOC), enveloped by budding through the endoplasmic reticulum, and then transported to the PM. Betaretroviruses (formerly called type B and type D retroviruses), for example Mason-Pfizer monkey virus (MPMV), pre-assemble an immature viral core near the MTOC, and the core itself is transported to the PM for envelopment there. These viruses are not considered further in this review. Rather, we focus on the most widely studied retroviruses in the genera called alpharetrovirus (e.g., Rous sarcoma virus, RSV in chickens), gammaretrovirus (e.g., murine leukemia virus in mice, MuLV), deltaretrovirus (e.g., human T-cell leukemia virus, HTLV), and lentiretrovirus (e.g., human immunodeficiency virus type 1, HIV-1). In fact, given the AIDS epidemic over the past two and a half decades and the extensive research it has spawned, more is known about HIV-1 than about any other retrovirus.

Immature retroviruses, i.e., before cleavage of Gag into its constituent domains by the viral protease, have a characteristic morphology as seen by thin section electron microscopy (EM). As early as the 1960s retroviruses were observed to bud from the PM of infected cells, and so the assumption reigned for many years that the PM was the site of assembly and budding. However, in the period ~2000–2005 an alternative model for budding was suggested for at least some viruses and some cell types [e.g., HIV-1 in macrophages, (Pelchen-Matthews et al., 2003) or MuLV in 293T and HELA cells (Sherer et al., 2003)]. According to this model, some or all virus budding occurs into a late endosome compartment, followed by fusion of that organelle with the PM, releasing the virus particles. This model was based in part on EM observations in HIV-infected macrophages. However, as it turned out, macrophages have enormously convoluted infoldings of the PM, making it impossible to define by EM alone if a membrane is the PM or contiguous with the PM, or is topologically distinct. Although there may be some exceptions, the present view of wild type (wt) HIV budding is that it occurs exclusively at the PM (Jouvenet et al., 2006; Deneka et al., 2007; Welsch et al., 2007). Thus, the budding sites originally interpreted to be internal in a macrophage cell actually appear to be in a specialized membrane compartment that is a deeply invaginated part of the PM (Deneka et al., 2007; Welsch et al., 2007). Similarly, in polarized T cells HIV budding takes place in a highly restricted part of the PM (Hogue et al., 2009; Llewellyn et al., 2010). These observations highlight the conclusion that Gag not only selects the PM, but in fact selects specific parts of the PM. The distinguishing features of these specialized regions remain to be elucidated. In principle such features could be proteins or lipids, or some aggregate property of these such as membrane phase behavior (see below).

## LIPIDS AND MEMBRANES

To better understand the role lipids play in Gag-membrane interactions and the formation of the viral envelope, it is necessary to have an understanding of lipid behavior. Lipids represent a diverse group of molecules involved in energy storage, signaling, and the structure of cellular membranes. Glycerophospholipids, characterized by a glycerol-based head group and two fatty acid chains, represent the predominant group of polar membrane lipids in eukaryotic cells (van Meer et al., 2008). The most common cellular glycerophospholipids are phosphatidylcholine (PC), phosphatidylserine (PS), phosphatidylethanolamine (PE), phosphatidylinositol (PI), and phosphatidic acid (PA). The sterol cholesterol is the most common non-polar membrane lipid.

The PM of cells is an asymmetric bilayer approximately 30 Angstroms thick and represents a minor percent of total cellular membrane lipids. Of the cellular proteins associated with the PM, only a fractions are transmembrane proteins. However, *in vitro* characterization of lipid mixtures has yielded many breakthroughs in the understanding of membranes, and so these studies are useful.

**FIGURE 1:**
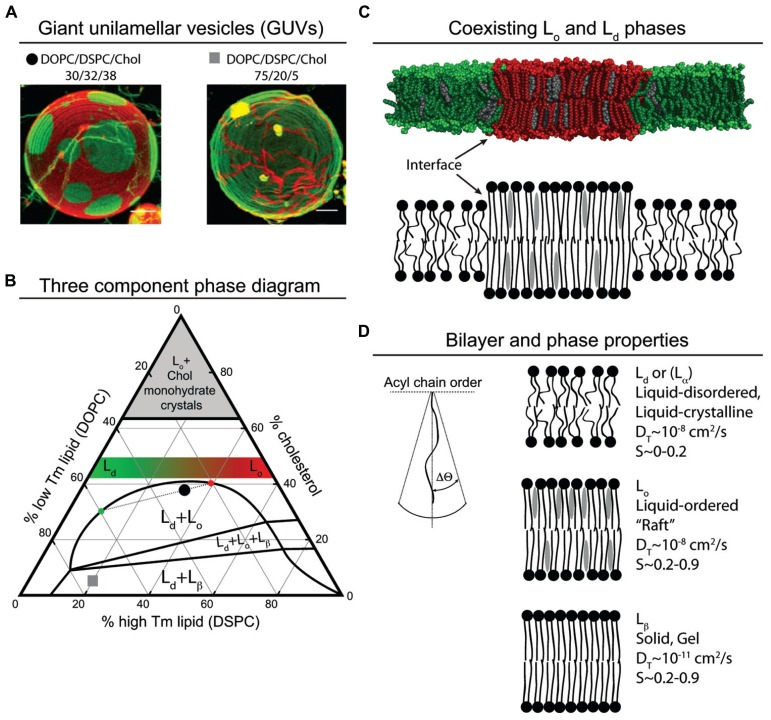
**Model membrane phase behavior and lipid parameters**. **(A)** Color merged GUV’s of simultaneously collected fluorescence emission from C20:0-DiI and (16:0, Bodipy)-PC reconstructed from confocal microscopy z-scans (Zhao et al., 2007). The red emitting C20:0-DiI segregates to both the L_o_ and L_β_ membrane phase, and the green emitting (16:0, Bodipy)-PC segregates to the L_d_ membrane phase. The lipid composition of each GUV is denoted by a black circle and a gray square on the phase diagram **(B)**. **(B)** The phase diagram is adapted from Zhao et al. (2007), Heberle et al. (2010) and Konyakhina et al. (2013). The vertices of the triangle represent pure components: 100% cholesterol, 100% low Tm lipid dioleoyl-PC (DOPC), and 100% high Tm lipid distearoyl-PS (DSPC). Each side of the triangle represents a binary mixture. The left side of the triangle represents lipid mixtures resulting in L_d_ membranes, and the right side of the triangle represents lipid mixtures resulting in L_o_ membranes. As the low Tm lipid DOPC is replaced with the high Tm DSPC at a fixed cholesterol concentration, for example, above 42% the membrane phase becomes increasingly ordered as depicted by the green to red gradient trapezoid. The one phase region falls outside of the labeled two and three phase regions. Lipid mixtures that fall in the region of the phase diagram labeled with more than one phase, for example the black circle in the L_o_ + L_d_ region, result in membranes with co-existing phases. The dotted line through the black circle represents a tie line. The distance of the black circle from the phase boundaries along the tie line represents the percent of membrane that is L_d_ and L_o_, approximately 25 and 75%, respectively. The intersection of the tie line with the left or right boundary denotes the composition of the respective membrane phase. In this example, the intersection of the tie line on the left side of the boundary corresponds to an L_d_ membrane with an approximate composition DOPC/DSPC/Chol (60/10/30). The intersection of the tie line on the right hand side of the boundary corresponds to an L_o_ membrane with an approximate composition DOPC/DSPC/Chol (20/40/40). When preparing GUVs with a charged lipid such as PS, it seems reasonable to exchange a percentage of the PC lipid for PS lipid. Using the black circle as an example, if one replaces all the DOPC (30%) with DOPS (30%), the resulting GUV would have an L_d_ region with 60% PS and an L_o_ region with 20% PS. This simplified example of exchanging PC for PS does not take into account the possible effect that lipid head group has on membrane phase behavior. **(C)** Molecular dynamics snapshot of DOPC/DSPC/Chol lipid segregation in a membrane bilayer. Line tension drives the formation of macro domains, due in part to differences in membrane thickness; L_o_ is thicker than L_d_. Because the interface is costs energy, in some lipid mixtures, micro domains coalesce into macro domains to minimize the interface. **(D)** Properties of different membrane phases and acyl chain order (adapted from van Meer et al., 2008).

Lipids can be characterized by a number of measurements including their rate of lateral diffusion (translational diffusion coefficient, D_T_) and the order, or range of movement, of their acyl chains (S). Defining the characteristics of lipids in a membrane bilayer can also be thought of as defining the phase behavior of the membrane bilayer. Phase behavior refers to the state of motion and order of individual lipids and how this state changes as temperature or composition change (van Meer et al., 2008). Membrane phases include liquid-disordered (L_d_), liquid-ordered (L_o_), and solid-gel (L_β_; **Figure 1**). L_d_ lipid bilayers generally have lower concentrations of cholesterol and higher concentration of lipids with unsaturated acyl chains (van Meer et al., 2008). The lipids are loosely packed with their chains sampling a large cone of space below the lipid’s head group. This cone of space that the acyl chain samples can be represented by a ΔΘ (**Figure 1D**). Due to the thin but wide volume of the lipid chains, the L_d_ bilayer is thin. L_β_ bilayers are composed mainly of saturated lipids that pack close to each other and have a low ΔΘ (van Meer et al., 2008). Additionally, the rate that lipids exchange with each other in the L_β_ bilayer is at least a 1000-times slower than in L_d_ bilayers (van Meer et al., 2008). L_o_ bilayers typically contain a mix of saturated and unsaturated acyl chains and cholesterol, and while the bilayer has a ΔΘ similar to that of L_β_, its D_T_ is similar to that of liquid disordered phase (van Meer et al., 2008).

Similar to lipid acyl chains, cholesterol must be shielded from the aqueous environment, but unlike phospholipids cholesterol has only a tiny hydrophilic head group. Shielding of cholesterol in the bilayer is accomplished by straightening of lipid acyl chains (a decrease in ΔΘ) so that the lipid head group can shield both its own acyl chains and cholesterol (Huang, 2009).

Membrane phase behavior can be determined for mixtures of up to three- and four-components. These data can be displayed in a phase diagram. A typical three-component diagram displays the membrane phase behavior for mixtures of cholesterol, a high Tm (saturated) lipid, and a low Tm (unsaturated) lipid. These diagrams can be used to determine if a given mixture forms one, two, or three phase, and if those phases are L_o_, L_d_, or L_β_. Phase diagrams also define the composition of mixtures that are near critical points between two-phase and one-phase mixtures.

Membrane phases can be nanoscopic or microscopic. Nanoscopic domains are typically characterized using Förster resonance energy transfer (FRET) while macroscopic domains can be observed with an optical microscope by employing giant unilamellar vesicles (GUVs) prepared with fluorescent dyes that partition into different phases (Heberle and Feigenson, 2011). FRET also measures the partitioning of lipid dyes in bilayers (Buboltz, 2007). An advantage of FRET is that preparation of the membranes by rapid solvent exchange (RSE) does not require lipids to transition through a dry phase, a step that is required in GUV preparation that may introduce artifacts (Buboltz and Feigenson, 1999). The use of GUVs to study membrane phase behavior yields striking and convincing evidence for the presence of lipid phases (**Figure 1**).

Formation of distinct, large and visible membrane phases has never been observed in living or fixed cells without extensive crosslinking, but this does not mean that phase coexistence do not occur. The phases can be nanoscopic and so not observable by microscopic techniques. There is indirect evidence for the existence of phase coexistence behavior in cellular membranes. For example, treating cells with cold detergent results in the isolation of a highly ordered, raft-like lipid and protein fraction termed detergent resistant membranes (DRMs). DRMs are enriched in cholesterol and sphingolipids (Brown and Rose, 1992; Simons and Ikonen, 1997) and one study found enrichment of arachidonic acid (20:4) and PE (Pike et al., 2002). In another study, electron spin resonance (ESR), a method that measures the order of lipid bilayers, showed that DRMs isolated from rat peripheral blood cells (RBL-2H3) were liquid ordered (Ge et al., 1999). While the behavior of DRMs has convincingly been defined as raft-like, it remains unclear to what degree DRMs represent a phase present in living cells or alternatively represent an artifact generated during cold detergent treatment of cells (Brown, 2006).

Further evidence for cell membrane phase behavior comes from microscope-based studies that characterize GUV-like membranes isolated from the PM of cells. The first study collected giant PM-derived vesicles (GPMV) from cells by treating cells with paraformaldehyde, which induces membrane blebbing (Baumgart et al., 2007). At a range of temperatures below 25°C GPMVs demonstrated lipid dye partitioning similar to that observed for model membranes with optically resolvable L_o_ and L_d_ phases (Baumgart et al., 2007). In a second study GPMVs were collected by the paraformaldehyde method and also by an alternative method that involves osmotic swelling of plasma membrane spheres (PMS) with PBS buffer (Kaiser et al., 2009). The PMSs demonstrated phase separation similar to GPMVs, and the inclusion of a PM protein, the transmembrane protein linker for activation of T cells (LAT) in the L_o_ phase, also was detected (Kaiser et al., 2009). The L_o_ phase of PMSs is significantly less ordered than that of the model membrane used for comparison. One explanation for the difference in order is the difference in cholesterol content; the GUVs were prepared with 20% cholesterol while the PMSs may have had as much as 45% (Kaiser et al., 2009). In addition to differences in cholesterol content compared to the GUVs, the GPMVs and PMSs were treated with cholera toxin to induce phase separation (Hammond et al., 2005).

## NATURE OF RETROVIRAL MEMBRANES

Like all natural membranes, retroviral membranes are made of lipids and proteins. The protein components prominently include the products of the *env* gene, i.e., trimers of the SU-TM complex that is formed in the Golgi. Minor amounts of cellular membrane and other proteins also are found in or associated with virions (Chertova et al., 2006), but to date most of these are without clearly known function. The lipid components of retroviral membranes were first analyzed systematically in the late 1970s and early 1980s for RSV (Quigley et al., 1971, 1972; Pessin and Glaser, 1980; it should be noted here that all alpharetroviruses appear to be strains of a single species, since they all show ~95% sequence identity, not counting viral oncogenes picked up from the cell, such as *src.* For convenience, in this review all of these strains are referred to as RSV). These early lipid analyses showed that the viral membrane was enriched in cholesterol and sphingolipids, compared with the PM. Similar results were found for HIV-1 and HIV-2 in the early 1990s (Aloia et al., 1993). A major limitation in interpreting these results is that the PM itself is difficult or impossible to obtain in pure form. Nevertheless, the retroviral composition seems to be enriched in the lipids that later were found to be enriched in membrane microdomains often referred to as “rafts” (Simons and Ikonen, 1997), and that also were found to be enriched in model membranes in the L_o_ phase (see below). This similarity gave rise to the over-simplified notion that retroviruses “bud from rafts.”

Consistent with the importance of rafts for retroviral budding are the results of cholesterol depletion from cells expressing HIV-1 or other Gag proteins. Such depletion can be accomplished by addition of methyl β-cyclodextrin (CD), which tightly binds cholesterol. How is CD, a highly soluble compound that does not enter a lipid bilayer, able to extract cholesterol from the hydrophobic environment inside the membrane bilayer? While at equilibrium cholesterol highly prefers to be in the membrane, according to one model at physiological temperatures cholesterol molecules may escape into the aqueous phase, where the cholesterol is captured by CD. Depletion of cholesterol from the PM is surprisingly rapid, in tens of minutes (Klein et al., 1995; Yancey et al., 1996), depending on the CD concentration. Addition of CD to HIV-1 Gag-expressing cells greatly reduces budding (Ono and Freed, 2001), apparently even in a time window and CD concentration range in which cell viability is not severely compromised. Nevertheless, given the importance of cholesterol for many cellular processes, interpretation of the results of CD depletion should be cautious. By *in vitro* liposome flotation assays, Gag binding is greatly stimulated by the presence of cholesterol, even under conditions where no L_o_ phase is present (Dick et al., 2012; see below).

Recently more accurate lipid compositions have been determined for HIV-1 by use of mass spectrometry (MS; Brugger et al., 2006; Chan et al., 2008; Lorizate et al., 2013). MS allows not only the lipid type as defined by headgroup to be identified (e.g., PS, PC, or PE) but also the composition of the many kinds of natural fatty acyl chains of each type, which vary in length as well as in the degree of unsaturation. As for earlier reports, a limiting factor in interpreting these results is the purity of the virus and the purity of the PM with which the viral membrane is compared. Several important generalizations emerge from these recent papers. First, HIV and MuLV membranes contain the phosphatidylinositol phosphate PI(4,5)P2, which has been inferred, at least for HIV-1, to be involved in Gag-PM recognition (Chan et al., 2008; see below). Second, retroviral membranes are enriched in PM outer leaflet lipids that are found in L_o_-like membrane microdomains (rafts), e.g., sphingomyelin. Third, HIV-1 is highly enriched in a lipid derived from PE, plasmalogen PE, which comprises a major species of PE in the PM (Chan et al., 2008; Lorizate et al., 2013). The significance of the latter is uncertain. Retroviral membranes also are reported to be enriched in cholesterol, compared with the PM (Brugger et al., 2006; Chan et al., 2008). However, a more recent study did not find significant enrichment in cholesterol in the HIV envelope (Lorizate et al., 2013). Interpreting these results is limited by two factors. First, purification of virions away from cellular membranes is difficult. Second, as mentioned above, comparison of the lipid composition of viral membranes with that of the PM is limited by the lack of purity of the isolated PM fraction.

## PRINCIPLES GOVERNING THE ASSOCIATION OF Gag WITH THE PM

Several principles have been elucidated that underlie Gag targeting to the PM. First, the matrix (MA) domain of most retroviral Gag proteins is myristoylated. Myristate, a 14-carbon fatty acyl modification at an N-terminal Gly residue of proteins, is quite common in cellular proteins that interact with membranes, and in Gag proteins it is presumed to become inserted into the hydrophobic core of the bilayer. Gag myristoylation is essential for membrane binding (O’Carroll et al., 2012b), a conclusion that stems from the observation that mutation of the N-terminal Gly completely blocks budding and membrane localization of Gag; the Gly is known to be required for the N-myristoyl transferase that attaches this fatty acid to the nascent protein while it is still on the ribosome (Gordon et al., 1991). In the HIV MA domain as well as in some cellular proteins, the myristate can exist in a solvent-exposed state or in sequestered state in which the hydrophobic acyl chain lies in a pocket or groove in the protein (Tang et al., 2004). According to the “myristoyl switch” hypothesis (Ames et al., 1997), the solvent exposed state for monomeric MA is much less populated than the sequestered state, for example as shown by NMR (Tang et al., 2004). Thus the myristate-sequestered protein has low membrane binding potential. Myristate exposure can be triggered by multimerization (Tang et al., 2004) or by PI(4,5)P2 binding (Saad et al., 2006; see below), which in turns leads to membrane binding. One of the several *in vivo* observations that bolster the biochemical analyses *in vitro* is based on an MA deletion mutant form of HIV Gag. If Gag has no MA domain at all, but does have an ectopic exposed myristate just upstream of the CA domain, membrane binding and budding can occur (Borsetti et al., 1998; Reil et al., 1998).

While the myristoyl switch hypothesis is generally accepted, according to a recent molecular dynamic simulation, myristate exposure and subsequent membrane insertion can occur even without multimerization or PI(4,5)P2 binding (Charlier et al., 2014). The mechanistic implications of these results remain to be explored. Furthermore, a single fatty modification on a monomeric protein is known to be insufficient to stably lock a protein into a membrane (Silvius et al., 2006). In addition, some Gag proteins, like those of alpharetroviruses (e.g., RSV) and the lentivirus equine infectious anemia virus (EIAV), are not myristoylated at all. Both of these observations imply that retroviral Gag proteins also rely on other membrane binding signals.

A second principle for Gag-PM binding and budding is based on electrostatics. The MA domains of retroviruses share a high degree of structural homology. The N-terminal domain (NTD) of MA is composed of 5–6 major alpha helices that fold into a globular shape (Hill et al., 1996; Murray et al., 2005). The globular head of MA positions a number of basic amino acids on one surface resulting in a basic patch that is oriented towards the PM. This basic patch interacts electrostatically with the negatively charged inner leaflet of the PM. Most genera of retrovirus have such a basic patch, with the net surface charge of MA domains differing from neutral to +3 to +6, as in the case of EIAV, RSV, and HIV-1, respectively (Murray et al., 2005). The basic patch of HIV-1 MA also mediates binding of RNA, which may modulate electrostatic interaction of Gag with the PM (Alfadhli et al., 2009, 2011; Chukkapalli et al., 2010, 2013; Dick et al., 2013). To what extent this finding can be generalized to other retroviruses remains to be carefully explored.

The MA domain of RSV Gag is not myristoylated, and so RSV MA serves as a good model to probe electrostatic interactions between retroviral MA domains and membranes. Single and double mutations of basic to acidic amino acids in RSV MA result in a decrease or a loss, respectively, of Gag localization to the PM and of virion release (Callahan and Wills, 2000). In the background of a double basic to acidic mutant, mutations elsewhere in MA that return the net surface charge to +3 restore virion release (Callahan and Wills, 2000), indicating that the exact placement of the basic side chains in the structure is not important. Mutating two acidic amino acids to basic amino acids results in increased viral release (Callahan and Wills, 2000). This mutant Gag protein, dubbed super-M (super membrane binder, SM), fails to traffic through the nucleus of the cell, packages 1/10th of the viral genomic RNA (vgRNA) of wt particles, releases virus particles more rapidly than wt, and is non-infectious (Callahan and Wills, 2003). It remains unclear if the decrease in vgRNA packaging is due to the rapid viral release or to the defect in nuclear trafficking (Callahan and Wills, 2003).

Liposome flotation analyses of RSV MA and Gag strengthen the hypothesis thatits membrane binding is driven by electrostatics. RSV MA binding to liposomes composed of physiological amounts of the negatively charged phospholipid PS is decreased to undetectable levels as salt is increased from 75 to 500 mM NaCl (Dalton et al., 2005). A MA mutant with two basic lysine residues changed to the neutral asparagine is significantly defective in membrane binding (Dalton et al., 2005). Increasing the lipid concentration of PS increases the amount of protein that associates with liposomes (Chan et al., 2011). While some retroviral MAs bind specifically to PIPs, RSV MA has no known PIP binding pocket. However, RSV MA responds strongly to the presence of PIPs as measured by liposome flotation. This apparent discrepancy may be due to the high charge density of PIPs at physiological pH, and thus the observed increase in binding to membranes with PIPs may be electrostatic.

Compared with the net +3 charge of RSV MA, HIV MA has a net +6 charge. Interestingly, the first 31 residues of MA can function independently as a membrane-binding region, dependent on the basic residues and the myristate, as demonstrated by the membrane binding of a MA-Src chimera (Zhou et al., 1994). The MA-Src chimera likely does not maintain structural components required for forming the PIP binding pocket.

Similar to RSV MA, altering the number of basic residues in HIV MA alters HIV MA membrane binding. For example, mutation I18K/L20K results in nearly double the amount of membrane-associated Gag protein compared with wt as measured by flotation of postnuclear supernatants of virus expressing cells (Ono et al., 2000). The mutation K29T/K31T results in a three-fold reduction of virus release and K29E/K31E results in Gag accumulation at Golgi membranes in cells (Freed et al., 1995; Ono et al., 2000). Individual mutations of the basic residues K18, R20, and R22 each results in a dramatic decrease in viral infectivity. However, these mutants still produce virions but with lower levels of Env incorporation, which may account for the decrease in infectivity (Bhatia et al., 2007). Interestingly, mutations K29T/K31T and K29E/K31E do not significantly reduce binding to PC/PS membranes in a standard liposome flotation assay, but they do decrease binding to membranes that contain PI(4,5)P2 (Chukkapalli et al., 2008). Taken together these results show that electrostatics cannot solely explain the interaction of HIV-1 Gag with membranes.

A third principle, at least for some Gag proteins, such as HIV-1 and MuLV (Ono et al., 2004; Hamard-Peron et al., 2010; though perhaps not for all Gag proteins; Chan et al., 2011; Inlora et al., 2011) is the involvement of PI(4,5)P2 in MA interaction with bilayers. The Summers lab was the first to show that the HIV-1 MA domain binds specifically to water soluble (C4) and (C8) chain analogs of PI(4,5)P2 (Saad et al., 2006). Specific interactions between MA and PI(4,5)P2 was confirmed by protein footprinting (Shkriabai et al., 2006). Since this phosphoinositide is a marker for the PM, the specificity of binding *in vitro* was taken to imply that PI(4,5)P2 plays a critical role in HIV-1 Gag PM localization (Freed, 2006; Saad et al., 2006). This conclusion was supported by experiments in which PI(4,5)P2 was depleted in Gag-expressing cells, by means of a transfected 5-phosphatase, which resulted in abrogation of budding and relocalization of much of Gag to internal membranes (Ono et al., 2004). Also consistent with an important role for PI(4,5)P2 in HIV-1 budding was the finding that both HIV-1 and MuLV viral membranes incorporate relatively high levels of this phosphoinositide (Brugger et al., 2006; Chan et al., 2008). Reports of the influence of PI(4,5)P2 for RSV are inconsistent. We showed that depletion of PI(4,5)P2 *in vivo* did not significantly alter Gag PM localization or virus release, under the same conditions that HIV PM localization and budding were knocked down in parallel (Chan et al., 2011). However, using a more sensitive assay for changes in PM localization, the Parent lab demonstrated that PI(4,5)P2 depletion *in vivo* did reduce virus release (Nadaraia-Hoke et al., 2013). However, in that study HIV was not tested in parallel, and hence plausibly RSV is less sensitive to PI(4,5)P2 depletion than HIV. *In vitro*, we have reported that PI(4,5)P2 at low molar membrane concentrations does enhance binding of RSV Gag to membranes. Thus it remains unclear what role PI(4,5)P2 has for RSV Gag PM binding *in vivo*.

Much of the binding energy for PI(4,5)P2 and MA comes from hydrophobic interactions with acyl chains (Anraku et al., 2010), consistent with the observation that PI(4,5)P2 species with eight carbon-acyl (C8) chains bind much more tightly to HIV-1 MA than species with four carbon-acyl (C4) chains (Saad et al., 2006). From these several observations, a model emerged in which HIV-1 MA extracts (“flips out”) the sn2 acyl chain from hydrophobic environment in the PM and places it in a hydrophobic groove in the protein. Clearly from NMR results, this is what happens in the *in vitro* system based on shortened acyl chains. At the same time, the myristate chain normally sequestered in an MA pocket then “flips” into the membrane (Freed, 2006). This “flipping out” and “flipping in” model has gained wide currency. The model recently has been further elaborated with a report that not only for PI(4,5)P2, but also for PS, PC, and PE the sn2 acyl chains have specific binding sites on the surface of HIV-1 MA (Vlach and Saad, 2012).

Nevertheless, the “flipping “in/out” hypothesis remains an uncertain model for several reasons. Direct evidence for the specificity of binding *in vitro*, i.e., preference for PI(4,5)P2 over other phosphoinositides, rests on phospholipid molecules with short C4 and C8 chains, which are not found in biological systems. In quantitative assays for interaction of MA with liposomes containing biologically relevant phosphoinositides, little specificity for PI(4,5)P2 is observed (Chukkapalli et al., 2008; Chan et al., 2011). Furthermore, in liposomes approximating the composition of the inner PM leaflet, i.e., containing high levels of cholesterol and PS, HIV Gag binding is robust even without any phosphoinositides. The inner leaflet of the PM is highly negatively charged, and thus electrostatic interactions could account for much of the observed specificity toward the PM, as it does for proteins engineered to bind solely on the basis of charge (Yeung et al., 2008). For MuLV, the enhanced binding of MA to membranes by PIP2 was dependent on the presence of PS, suggesting that PIP binding is dependent on the net negative charge of the membrane from PS (Hamard-Peron et al., 2010). Finally, the published data on the effects of PI(4,5)P2 depletion by 5-phosphatase on retrovirus budding all are steady state measurements made many hours after the phosphatase has been expressed by transfection. Given the extremely dynamic nature of PIP pools, and the importance of PIPs for a multitude of cellular processes, it will always be difficult to interpret PI(4,5)P2 depletion experiments, unless systems are used which allow rapid and controlled recruitment of the phosphatase to the PM (Balla, 2007). Final acceptance of the flipping model probably will await crosslinking experiments or similar studies that directly detect that the sn2 acyl chain is no longer in the membrane but in the MA pocket.

A fourth and poorly understood factor in targeting of Gag to the PM is cellular trafficking. HIV-1 Gag, which is the best understood in this context, has binding sites for the clathrin adaptor proteins AP1 (Camus et al., 2007), AP2 (Batonick et al., 2005), and AP3 (Dong et al., 2005). Perhaps the best understood is the AP3 binding site in the MA domain. While ablation of this sequence does not completely abrogate PM binding and budding, it is reported to greatly reduce both of these readouts (Dong et al., 2005). According to a more recent report, MA does not directly interact with AP3, making interpretation of the earlier results difficult (Kyere et al., 2012). Thus, the mechanism by which adapter proteins actually work to promote PM interaction remains unknown.

Finally, a fifth principle that helps explain Gag-PM targeting is Gag multimerization. An intrinsic property of Gag is the propensity to multimerize, a process that is dependent of Gag concentration and on the interaction of NC with nucleic acid. At low concentrations, HIV Gag with a dimerization constant of 1–10 mM (Gamble et al., 1997; Datta et al., 2007b) is found to be largely cytoplasmic (Fogarty et al., 2013). As the concentration of Gag increases in the cytoplasm the fraction of Gag found at the PM increases, likely because Gag multimerization increases (Fogarty et al., 2013). In the cytoplasm the predominant fraction of Gag is monomeric with a subfraction being dimeric (Kutluay and Bieniasz, 2010). Consistent with the model that the PM induces Gag assembly, there is an enrichment of higher multimeric complexes of Gag at the PM (Kutluay and Bieniasz, 2010).

Gag interaction with the PM presumably also promotes Gag multimerization. Knocking out HIV Gag membrane association by preventing myristoylation, by mutating the terminal glycine residue, results in a loss of viral particle assembly (O’Carroll et al., 2012b). Nevertheless, at sufficiently high concentrations of Gag, assembly is detected in the cytoplasm, independent of Gag membrane association (O’Carroll et al., 2012a, b).

Fluorescent correlation spectroscopy (FCS) measurements of cytoplasmic RSV Gag-GFP movement show that in the RSV system, Gag is in large complexes prior to localization to the PM (Larson et al., 2003). However, these complexes contain only a few Gag proteins (Larson et al., 2003), implying the involvement of cellular proteins, perhaps like those implicated in HIV assembly (Lingappa et al., 1997). FRET measurements of RSV Gag-YFP and Gag-CFP show that Gag-Gag association occurs prior to membrane binding of Gag.

Fusion of the Gag membrane binding domain, MA, to proteins with defined multimerization states allows for a measurement of the effect of multimeric state of MA on membrane binding, both *in vivo* and *in vitro*. For example, for HIV MA, dimerization enhances membrane binding *in vitro* and *in vivo* (Dalton et al., 2005; Dick et al., 2013). While for RSV MA dimerization is not sufficient to promote substantial PM binding *in vivo*, hexamerization leads to strong PM association (Dick et al., 2013). Overall, these findings suggest that Gag multimerization is an early, critical step in the association of Gag with the PM in cells.

Taken together these results are consistent with a model in which early steps of Gag multimerization occur prior to membrane localization. Once these small Gag multimers associate with the membrane, additional Gag molecules rapidly associate with the Gag multimer resulting in the assembly of a virus particle. Because at typical Gag levels, assembly into a complete virion does not occur in the cytoplasm, the PM must provide a feature required for assembly. This feature may be a restrictive (2 dimensional) environment with locally high concentrations of Gag compared with cytoplasm. Additionally, or alternatively, the PM may provide a critical component that promotes multimerization, such as a cellular protein, a lipid, or a membrane domain.

The discussion above, like most published discussions of Gag-membrane interaction, focus on the N-terminal MA domain, since it is the known module that is able to bind membranes *in vivo* and *in vitro*. However, some evidence suggests that the NC domain also may play a role in membrane interaction, at least in the case of HIV-1.

Size exclusion chromatography (SEC) and small angle X-ray scattering (SAXS) of HIV-1 Gag show the Gag polyprotein to be in a compact, horseshoe conformation (Datta et al., 2007a). This conformation is also observed for dimers of Gag (Datta et al., 2011). These conformation studies raise the possibility that both the MA and NC domain interact with the cellular PM prior to or during virion assembly. Consistent with the SEC and SAXS data, low angle neutron reflectometry (LANR) studies of HIV-1 Gag on a supported bilayer show Gag is compact, and both the MA and NC domains interact with the supported bilayer (Datta et al., 2011). The addition of a short oligonucleotide, too short to induce assembly of Gag into VLPs, results in the extension of the Gag protein (Datta et al., 2011). This extended structure is interpreted as MA bound to the membrane and NC bound to the oligonucleotide (Datta et al., 2011). Taken together these observations have led to a model in which both ends of Gag bind to the PM. Upon the binding of vgRNA to NC, which outcompetes NC binding to the PM, Gag takes on an assembly competent extended conformation. A limitation of these studies is that the experiments were done with non-myristoylated Gag. Further evidence will be needed to assess this model critically.

## ASSAYS FOR Gag-MEMBRANE INTERACTION AND CHALLENGES IN INTERPRETING THE RESULTS

Many methods have been used to study Gag-membrane interactions, each with its own limitations. Quantitative parameters are particularly challenging to elucidate. In overview, the relevant experiments can be grouped into two classes: *in vivo* studies with transfected or infected cells, and *in vitro* studies with purified or specifically labeled proteins and artificial or natural membranes.

### Gag-PM LOCALIZATION *IN VIVO*

The most straightforward and very common method to visualize Gag subcellular localization is fluorescence microscopy of GagGFP fusions (or more rarely, immunofluorescence). With some exceptions, such studies typically give information on the steady state distribution of Gag, and do not report on Gag trafficking from the site of synthesis in the cytoplasm to the PM, or to other locations. At steady state the PM is enriched in Gag, but the cytoplasm also has abundant levels of Gag, depending somewhat on the species of retrovirus (Fogarty et al., 2013).

### Gag-MEMBRANE INTERACTION *IN VITRO*

Probably the most standard method to visualize Gag or MA interaction with membranes *in vitro* is liposome sedimentation or flotation. Liposomes that have bound protein are either pelleted by high speed centrifugation, or alternatively after addition of sucrose are floated upwards through a sucrose step gradient, also by high speed centrifugation. The former version is less robust because aggregated protein also will pellet to the bottom of the tube. These techniques are low throughput, requiring one gradient to obtain one data point.

Liposome flotations are relatively easy to set up, involving the preparation of uni- or multi-lamellar vesicles (ULVs and MLVs, respectively), both of which will float to the top of a sucrose gradient. Preparation of vesicles typically involves the combination of two or more lipids at defined molar concentrations in an organic solvent, in most cases chloroform. The lipid mixture is dried down to a film and resuspended in an aqueous buffer. Depending on the concentration and composition of the lipid mixture, the resuspended lipids may require multiple freeze thaw cycles to fully rehydrate the film. At this stage the lipids are in multi-layered (multi-lamellar) membrane vesicles ranging in size from 100 to 1000 nm. Because the vesicles are multi-lamellar, it is not possible to predict the area of membrane that is available to bind proteins. In addition, the concentration of each lipid in the outer layer of membrane may not be the same as in the inner layer. For example, in a MLV charged lipids like PS or PIPs may preferentially segregate to the outermost layer that is exposed to the aqueous environment, resulting in an effectively higher concentration of the charged lipid. Repeated extrusion of MLVs through a membrane of defined pore size results in ULVs with a defined range of size (about ±20 nm for 100 nm pores), and thus known concentrations of lipid are available to surface-binding proteins.

One disadvantage of preparing MLVs and ULVs by the dry film method is that at high concentrations (the exact concentration varying depending on the lipid mixture) cholesterol forms monohydrate crystals when the organic solvent is removed during the drying process. The cholesterol crystals may not fully rehydrate, resulting in a final membrane with a lower than the intended cholesterol concentration. RSE was developed to overcome the cholesterol monohydrate crystal artifact (Buboltz and Feigenson, 1999). RSE takes advantage of the low vapor pressure of organic solvents compared with the vapor pressure of aqueous buffers. When lipids dissolved in organic solvent are mixed with an aqueous buffer under a vacuum the organic solvent evaporates and the lipids are effectively transferred to the aqueous buffer. RSE of lipids results in a mixture of ULVs and MLVs in the range of 100 nm in diameter (Buboltz and Feigenson, 1999).

Liposome flotation can be performed with crude or purified protein. Using purified protein provides a greater amount of control over protein concentration and buffer conditions of the protein-membrane binding reaction. Dalton et al. (2007) found that binding of RSV and also HIV MA protein to liposomes is largely dependent on the ionic strength of the buffer. Additionally, they showed that for both viruses MA dimerization results in at least an order of magnitude increase in tightness of liposome interaction (Dalton et al., 2005, 2007). Purified protein-based flotations have also been used to show that increasing the multimeric state of RSV MA to a hexamer (as previously mentioned) results in a large increase in affinity for liposomes (Dick et al., 2013). Also, RNA can inhibit binding of purified RSV and HIV MA to liposomes, but this result is dependent on low (50 mM NaCl) ionic strength (Dick et al., 2013).

Some proteins are not tractable in the bacterial expression systems typically used for generating protein. For example, purification of myristoylated HIV Gag in concentrated form is difficult or impractical. An alternative method largely pioneered by the Ono lab (Chukkapalli et al., 2008) is the generation of 35SMet labeled protein by *in vitro* translation in a commercially available reticulocyte extract. Advantages of this system include the presence of physiological concentrations of protein, nucleic acid, and ions, as well as efficient myristoylation, which is essential for HIV Gag-membrane interaction. However, *in vitro* translation is not without its downsides. Most important, in assays such as membrane binding, it is very difficult to sort out the possible confounding effects of cellular proteins, RNA, or metabolites. In addition, because of the high concentrations of nucleic acid in the reticulocyte reaction, Gag proteins that are induced to assemble in the presence of nucleic acid may not be monomeric, or may be aggregated. Not knowing the multimeric state of the protein makes interpretation of membrane binding results difficult in light of the importance of multimerization on MA’s interaction with membranes.

To visualize by fluorescence microscopy the interaction of Gag with membranes of defined lipid composition, standard liposomes obviously are not suitable because of their small size. Instead, for this purpose GUVs can be used. GUVs are a common tool to study the phase behavior of lipids. Because GUVs are so large (in the range of 10–50 microns in diameter) they allow real time analysis of the binding of fluorescently tagged proteins. For example, GUVs were used to study the binding of ESCRT proteins, known to be involved in viral budding, to HIV-1 Gag assembly sites (Carlson and Hurley, 2012). Taking advantage of the observable phase behavior of GUVs, Keller et al. (2012) studied the binding of artificially multimerized HIV MA to GUVs having both L_o_ or L_d_ membrane domains. The multimeric MA was found to preferentially to the L_d_ domain. However, the interpretation that MA favored L_d_ because of the lipid phase itself in this case is not persuasive because the relative partitioning of acidic lipids into the L_o_ and L_d_ is not known.

A major hurdle of using GUVs to study electrostatic protein-membrane interactions is that phase diagrams for PS- and PIP-containing lipid mixtures do not exist. This makes the interpretation of results generated using lipid mixtures containing PS and PIP difficult. Lipid head groups influence lipid phase behavior, and so one cannot make the assumption that replacing some fraction of a PC lipid with a PS lipid will leave the phase behavior unchanged. The uncertainty of lipid phase behavior in PS mixtures can be compounded by the addition of PIPs such as PI(4,5)P2. Under some buffer conditions and in some lipid mixtures, PIPs do not mix with other lipids, instead forming their own phase (Redfern and Gericke, 2005; Kooijman et al., 2009; Wang et al., 2012). Not knowing what the behavior is of PIPs in membranes is a major limitation for correctly interpreting experimental results for PIP-containing mixtures.

Another difficulty in working with GUVs is their sensitivity to osmotic pressure. Because the electrostatics of Gag binding to membranes is so heavily influenced by ionic strength, it is important to choose buffer and salt conditions that are biologically relevant. Unfortunately, preparation of GUVs is significantly influenced by the ionic strength of the solution. Typically GUVs are made in the absence of ions or at very low ionic strength. Matching the osmotic strength of the GUVs with the osmotic strength of the protein and at the same time maintaining a physiological ionic strength takes careful planning.

## PERSPECTIVES

Key principles that direct the binding of Gag to membranes include electrostatics, fatty acid modification of MA, multimerization of Gag, interaction with lipids such as PI(4,5)P2, and the ability of MA to sense the hydrophobic core of the membrane. However, much remains to be learned about these key signals. What are some of the critical issues?

While we know that HIV-1 Gag membrane binding is sensitive to the presence of PI(4,5)P2 in membranes (Ono et al., 2004; Chukkapalli et al., 2008; Chan et al., 2011), and that *in vitro* as little as 2% PI(4,5)P2 can increase Gag membrane binding by 10-fold (Dick et al., 2012), we understand surprisingly little about how PIPs behave in membranes. Evidence is emerging from studies of model membranes that PIPs cluster in membranes (Redfern and Gericke, 2005; Kooijman et al., 2009). Strong intermolecular and intramolecular hydrogen bond networks drive the formation of the PIP clusters, and under certain conditions these clusters represent a separate membrane phase (Redfern and Gericke, 2005; Kooijman et al., 2009; Wang et al., 2012). These PIP clusters might serve as specialized binding sites for retroviral Gag proteins. If PIP clustering proves to be a biologically relevant type of lipid organization, characterizing it will be extremely important.

Many Gag-membrane binding studies have been based on lipid mixtures that are not representative of the lipids found in the inner leaflet of the PM. Therefore, developing a model inner leaflet lipid mix and studying the properties of this mix should be a priority. For example, model membranes are typically made with the neutral outer leaflet lipid PC instead of the inner leaflet lipid PE. PC and PE have large differences in head group size, which could dramatically affect protein binding. In addition, cholesterol is frequently not included in model membrane mixes, nor are lipids that represent smaller fractions of the membrane such as sphingomyelin, plasmalogen-PE, and PI. Not only is a model inner leaflet lipid mix rarely used to study protein-membrane interactions, but essentially no information is available on the phase behavior of such a lipid mixture. Efforts should be made to characterize biologically relevant lipid mixtures.

The binding of any protein to a membrane involves a limited number of principles. So the study of one protein-membrane interaction may shed light on how other proteins interact with membranes. Therefore future work in the field of retroviral protein membrane binding may have broad implications to cellular biology.

## Conflict of Interest Statement

The authors declare that the research was conducted in the absence of any commercial or financial relationships that could be construed as a potential conflict of interest
